# A Simple, Sensitive and Safe Method to Determine the Human α/β-Tryptase Genotype

**DOI:** 10.1371/journal.pone.0114944

**Published:** 2014-12-29

**Authors:** Quang Trong Le, Sahar Lotfi-Emran, Hae-Ki Min, Lawrence B. Schwartz

**Affiliations:** Department of Internal Medicine, Virginia Commonwealth University, Richmond, Virginia, United States of America; University of Florida, United States of America

## Abstract

The human tryptase locus on chromosome 16 contains one gene encoding only β-tryptase and another encoding either β-tryptase or the homologous α-tryptase, providing α:β gene ratios of 0∶4, 1∶3 or 2∶2 in the diploid genome, these genotypes being of potential clinical relevance in severe atopy. Using an EcoRV restriction site in α- but not β- tryptase, PCR products, spanning intron 1 to exon 5, were used to determine α/β-tryptase gene ratios using non-radioactive labels, including ethidium bromide labeling of all PCR products, and either digoxigenin-primer or DY682-primer labeling of only the final PCR cycle products. Sensitivity increased ∼60-fold with each final PCR cycle labeling technique. Ethidium bromide labeling underestimated amounts of α-tryptase, presumably because heteroduplexes of α/β-tryptase amplimers, formed during annealing, were EcoRV resistant. In contrast, both final PCR cycle labeling techniques precisely quantified these gene ratios, because only homoduplexes were labeled. Using the DY682-primer was most efficient, because PCR/EcoRV products could be analyzed directly in the gel; while digoxigenin-labeled products required transfer to a nitrocellulose membrane followed by immunoblotting. This technique for determining the α/β-tryptase genotype is sensitive, accurate, simple and safe, and should permit high-throughput screening to detect potential phenotype-genotype relations for α/β-tryptases, and for other closely related alleles.

## Introduction

The human tryptase locus on chromosome 16 includes genes that encode α- and β- tryptases [1], which are major protein products of human mast cells, lesser amounts being expressed by basophils, and none being expressed by other cell types [2]. The locus includes one gene that always expresses a β-tryptase (TPSB2), and another one that can express either α- or β- tryptase (TPSAB1). Thus, α:β tryptase gene ratios are typically 0∶4, 1∶3 or 2∶2, depending on whether the parental TPSAB1 genes encode either α- or β- tryptase. Importantly, mature β-tryptase is proteolytically active as a homotetramer, whereas mature α-tryptase is proteolytically inactive, despite having 93% protein sequence identity to β-tryptase [3]–[6], and pro and mature tryptase serve as biomarkers of human diseases such as mastocytosis and systemic anaphylaxis [2]. A frame-shift mutation in the β-tryptase gene, though uncommon, also has been detected at TPSB2 in Caucasians and African Americans, but not in Asians, giving rise to a truncated inactive form of β-tryptase, and when present, only full length β-tryptase has been detected at the TPSAB1 locus, seemingly ensuring that everyone expresses an active form of β-tryptase [7]. This locus also includes TPSD1, the δ-tryptase gene [1], originally called MMCP-7-like gene, harboring a mutation that truncates the protein 40 amino acids short of 245 amino acid sequences of the mature α/β-tryptases, likely making this a pseudogene [1], [8], [9], though one report contests this conclusion [10]. TPSG1, the γ-tryptase gene, encodes a transmembrane protease that is quite distinct from the α/β-tryptases [11], [12]. Measuring allelic α/β-tryptase gene ratios can be a powerful approach toward detecting potential involvement of α-tryptase on disease phenotypes. For example, in one study the 2∶2 but not 1∶3 α:β tryptase genotype, compared to the 0∶4 α:β genotype, was associated with higher serum levels of IgE and more severe atopy [13].

PCR-sequencing was used initially to demonstrate that 20% of Caucasians and African Americans, and 10% of Asians were α-tryptase gene deficient, but incorrectly concluded these deficiencies were due to a deletion of the α-tryptase gene rather than allelic heterogeneity and did not attempt to distinguish between 2∶2 and 1∶3 α:β tryptase genotypes [14]. One strategy distinguishing α- from β- tryptase alleles utilizes restriction fragment length polymorphism methods. In particular, an EcoRV restriction site in α-tryptase genomic DNA (gDNA)^1^ was identified [15], that was later utilized to distinguish α (cleaved) from β (uncleaved) tryptase gene PCR products [16], allowing the α:β tryptase gene ratios (genotype frequency) to be calculated at 2∶2 (0.21), 1∶3 (0.50) or 0∶4 (0.29), and again finding that α-tryptase gene deficiency was higher in Caucasians (0.45) and African Americans (0.26) than Asians (0.13). However, the use of ethidium bromide to measure PCR amplimers and their cleavage products provided potential ambiguity in distinguishing 2∶2 from 1∶3 α:β tryptase gene ratios, because α/β-tryptase gene amplimers may form heteroduplexes that resist EcoRV cleavage, increasing the apparent amount of the β-tryptase gene. This potential limitation was overcome using a hot-stop technique, introducing radioactive nucleotides only during the final PCR cycle, such that all labeled PCR products were homoduplexes [17], and yielding α:β tryptase gene ratios (frequencies) of 2∶2 (0.29), 1∶3 (0.44), and 0∶4 (0.26). However, radioactive agents are inherently hazardous. Another technique used for quantitative tryptase genotyping involved performing a high resolution melt curve on a 70 bp PCR product from Exon 4, having 6 nucleotide α:β mismatches [13]. This study reported an α-tryptase gene deficiency of 57%, a genotype with 1 α-tryptase gene in 31%, 2 α-tryptase genes in 11%, and 3 α-tryptase genes in 1%, but utilized primers that recognize δ-tryptase as well as α- and β- tryptase genes. The δ-tryptase amplimer, having 3 nucleotide mismatches with α-tryptase and 5 with β-tryptases, may have affected the apparent α/β genotype results calculated from the melt curves.

Using nonradioactive labelled primers not only eliminates the hazards of working with radioactivity, but also may increase resolution and sensitivity [18], [19]. We report a novel non-radioactive PCR genotyping method for efficiently and accurately genotyping the ratios of α- and β- tryptase genes in genomic DNA, utilizing a 3′-primer, conjugated with an infrared-fluorescent label, a technique that should be broadly applicable to quantification of other duplicated genes with minor sequence differences. IRD-700 or DY862 label detection at infrared wavelengths provides high sensitivity, in part due to the very low background of infrared auto-fluorescence, being more sensitive than visible fluorescence [19]. Furthermore, these dyes have excellent water solubility, minimal nonspecific binding, and do not interfere with PCR. Compared to the direct IRD fluorescent PCR method, using digoxigenin-labeled DNA primers for PCR, detected with IRD-700-labeled anti-digoxigenin IgG, is very sensitive and exhibits low levels of nonspecific binding compared to other techniques [20], [21].

Our new non-radioactive PCR/restriction enzyme digestion method for determining allele-specific quantitative gene ratios offer several advantages over other previous methods, including: (i) high sensitivity; (ii) ease of performance; (iii) safety; and (iv) a stable labeled primer, and was used to determine the α:β-tryptase genotypes in genomic DNA from 24 individuals.

## Materials and Methods

### Reagents

Betaine (B0300), Me_2_SO (DMSO), glycerin, phosphate-buffered saline, 2-(*N*-morpholino)ethane sulfonic acid (MES), Hepes, Tris, EDTA, bovine serum albumin, agarose, and MgCl_2_ (Sigma-Aldrich Chemical Company LLC, St. Louis, MO); dNTP mix, gDNA purification kit, RNase-free DNase, and EcoRV (Promega Company, Fitchburg, WI); ProbeQuant G-50 Micro column (Amersham, Piscataway, NJ); FastStart Taq DNA Polymerase (Roche Diagnostics, Indianapolis, IN); polyacrylamide gels (Invitrogen, Carlsbad, CA); goat affinity-purified polyclonal IgG anti-digoxigenin (Vector Laboratories, Burlingame, CA); and IRDye680RD-labeled affinity-purified donkey IgG anti-goat IgG (LiCor, Lincoln, NE) were obtained as indicated.

Human mast cell leukemia cells (HMC)-1,obtained from a patient with mast cell leukemia and provided by Dr. G. Gleich and Dr. J. Butterfield (Mayo Clinic, Rochester, MN) [22], known to contain only βI and βIII variants of the β-tryptase gene; [7] and Mono Mac-6 cells[23], a cell line established from a patient with monoblastic leukemia and containing both α- and β- tryptase genes [24], were cultured in RPMI 1640 medium supplemented with 10% heat-inactivated fetal calf serum, L-glutamine, penicillin and streptomycin at 37°C/5% CO_2_. Primary human skin mast cells (SMCs), obtained from discarded surgical skin of 24 subjects (10 African Americans, 14 Caucasian or Latino), who ranged in age from 21 to 60 years, with written informed consent as approved by the Virginia Commonwealth University Internal Review Board, were cultured in serum-free X-Vivo-15 medium (Lonza Inc., Allendale, NJ) containing recombinant human SCF (100 ng/ml)(provided by Swedish Orphan Biovitrum, Stockholm, Sweden) as described [25].

Genomic DNA from SMCs, HMC-1, or Mono Mac-6 cells was purified using the Wizard Genomic DNA Purification Kit (Promega, Madison, WI). Oligonucleotides were synthesized and sequenced by the Nucleic Acid Core facility at Virginia Commonwealth University. DY682 (IRD700 replacement, MWG Operon, Huntsville, AL), [19] and digoxigenin– (Integrated DNA Technologies, Coralville, IA) 3′-labeled primers, stored frozen (−20°C) at a 100 mM concentration in 10 mM Tris-HCl, pH 8.0, were obtained as indicated.

### PCR

Allele-specific PCR genotyping was performed using standard PCR and ethidium bromide labeling of the accumulated PCR product(s), or using DY682- or digoxigenin–labeled primers as described below. Briefly, an initial PCR was performed using unlabeled primers, the resultant product providing the template for the final PCR cycle, in which DY682- or digoxigenin- labeled primers were introduced. PCR was conducted with 2.5 ng of gDNA in a total volume of 25 µl, containing PCR reaction buffer with dNTP (200 µM), Taq DNA polymerase (0.75 U) and each primer (sense, 5′-GCCCTGCCCTGTGAGGCCC-3′; antisense, 5′-CCGTGTAGGCGCCAAGGTG-3′) (0.5 µM). These primers, as described [16], uniquely recognize α/β-tryptase genes, and do not recognize other tryptases, including δ- and γ- tryptases. The sense and antisense primers complement nucleotide sequences located in intron 1 and exon 5, respectively, and amplify a 1028 bp region from β-tryptase gDNA and a 1017-8 bp region in α-tryptase gDNA (due to a 10 or 11 bp deletion in intron 4), but an EcoRV restriction site resides only in the α-tryptase amplicon, being located in exon 4. EcoRV cleavage of the α–tryptase gene product yields fragments of 678 bp (5′ side, unlabeled) and 339–40 bp (3′ side, labeled). PCR using unlabeled primers begins with a denaturation step at 94°C for 5 min (one cycle), followed by 35 cycles consisting of 94°C for 30 sec, 60°C for 60 sec and 72°C for 60 sec. To eliminate α/β-tryptase heteroduplexes from the final labeled PCR product, the final PCR cycle was performed after DY682 3′-labeled or digoxigenin 3′-labeled primer was added to the PCR react mixture, thereby ensuring that only homoduplexes were labeled.

Human mast cell tryptase genes are GC-rich (67–68%), making its amplification more difficult. The use of several additives during PCR was explored to improve amplification, including betaine, DMSO, glycerol and ethylene glycol. These agents were diluted in water and added in varying concentrations to PCR reaction mixtures, final concentrations being 1–10% for DMSO or 0.5–2.5 M for the others.

### Restriction enzyme digestion and analysis of PCR products

The PCR products derived from gDNA were digested with EcoRV (GAT**//**ATC recognition sequence) for 3–4 h, according to the manufacturer's instructions, and then subjected to electrophoresis in 1.2% agarose gels. For PCR genotyping, the fluorescent bands in the gel were directly imaged using the FluorChem^Tm^ Q system and analyzed with AlphaView SA software (ProteinSimple, Santa Clara, CA) after ethidium bromide labeling or the Odyssey system (LI-COR Biosciences, Lincoln, NE) after labeling with the DY682-tagged 3′-primer. After PCR using the digoxigenin-labeled primer, DNA products were transferred to a positively charged nylon membrane using a semi-dry transfer cell (Bio-Rad Laboratories Inc., Hercules, CA) under a constant amperage (50 mA, 25 V max for 1 h). The nylon membrane was then baked in a vacuum to link the DNA to the membrane, blocked for 1 h at room temperature in 2x Licor blocking reagent, and then incubated with goat polyclonal anti-digoxigenin IgG at 1∶1000 dilution for 2 h. After washing, the membrane was incubated with IRD700-conjugated secondary donkey IgG anti-goat IgG at a 1∶10,000 dilution for 30 min before being washed, imaged and analyzed on the Odyssey system. Neither DY682- nor digoxigenin- labeled product appeared to inhibit EcoRV restriction endonuclease activity.

### DNA sequencing

Sequencing of PCR products was used to detect allelic differences. The presence of α- and β- tryptase genes in cell lines was analyzed in PCR products obtained after 35 cycles of 94°C for 30 sec, 58°C for 60 sec, and 72°C for 60 sec as described above using non-labeled primer sets. The PCR products were analyzed by direct sequencing with a BigDye Terminator Cycle Sequencing Kit (Life Technologies, Grand Island, NY) on an automated sequencer, the 3130xl Genetic Analyzer (Applied Biosystems, Life Technologies).

### Statistics

Data groups followed a normal distribution and were analyzed by either a student's T test if only two groups were involved or ANOVA (all pairwise comparisons used the Holm-Sidak test) if there were more than two groups.

## Results

### Detection of α/β-tryptase genes in gDNA

**Figure 1:**
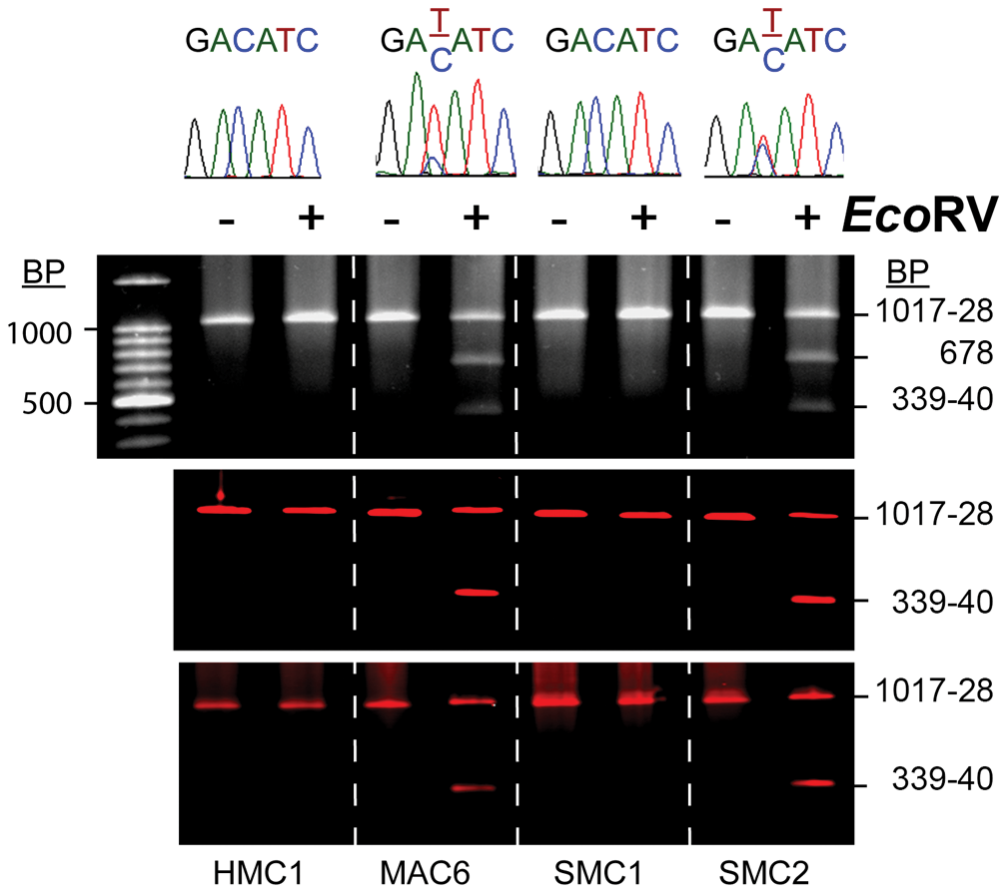
Standard and new PCR/restriction enzyme-based methods for α and β tryptase genotyping. Genomic DNA extracts from HMC-1, Mac-6 and two SMC preparations were each tested for the presence of β and α-tryptase genes by three different methods, each using the same 5′ and 3′ PCR primer pair as described in Methods. When the 1017-28 bp amplimers were exposed to EcoRV, the α-tryptase amplimer yielded 678 and 339-40 bp fragments. After labeling with ethidium bromide, all such products were detected (upper panel). In contrast, only the high molecular weight amplimer and 339-40 bp fragment were visualized using either DY682- (middle panels) or digoxigenin- (lower panels) labeled 3′-primer. DNA sequence analyses of the restriction site are shown above the corresponding gel electrophoresis pattern.

As expected, HMC-1 cell DNA after PCR/EcoRV yields a single band of 1017-28 bp (Fig. 1, all three panels), while Mac-6 cell DNA, known to have an αβ:αβ tryptase genotype (9), yields bands of 1017-28, 678 and 339-40 bp after ethidium bromide labeling (Fig. 1, upper panel) or bands of 1017-28 and 339-40 bp with DY682- or digoxigenin- labeled 3′-primer (Fig. 1, middle or lower panel, respectively), indicating that HMC-1 cells contain only the β-tryptase gene, while Mac-6 cells contain both α- and β-tryptase genes. DNA extracted from SMCs from two different subjects show only β tryptase DNA from SMC1, and both α and β tryptase DNA from SMC2. The presence of only β tryptase genes or of a mixture of α and β tryptase genes was confirmed by DNA sequencing, as indicated by the sequencing results above the panels in Fig. 1, showing the presence of the GATATC EcoRV sensitive α-tryptase DNA site along with the GACATC insensitive β-tryptase DNA site in the DNA PCR product from Mac-6 and SMC2 cell DNA, but only the GACATC insensitive site from HMC-1 and SMC1 cell DNA. These results demonstrate that the new nonradioactive PCR genotyping methods could be efficient tools for allele discrimination in genotyping.

### Sensitivity of α/β-tryptase gene detection by PCR

**Figure 2:**
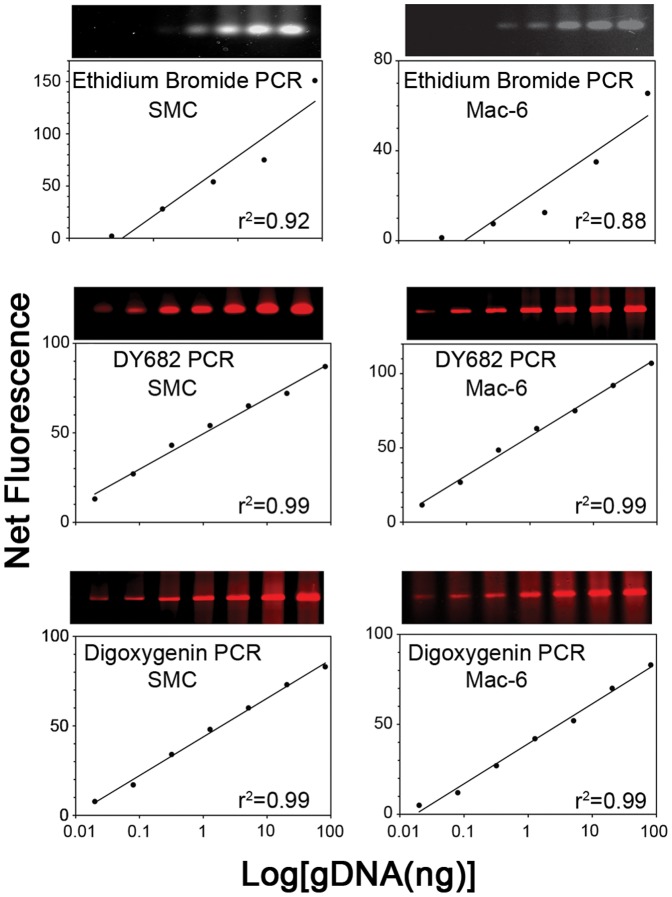
Sensitivity of standard ethidium bromide and new PCR amplimer detection methods for α/β-tryptase genes. Purified gDNA from a SMC preparation having both α- and β- tryptase genes and from MAC-6 cells, in each case at doses ranging from 0.02 to 81.9 ng, were subjected to PCR and labeled using ethidium bromide (top panels), or to PCR with DY682- (middle panels) or digoxigenin- (bottom panels) labeled 3′-primer. In each case labeled 1017-28 bp amplimer bands are shown along with the net fluorescence in the corresponding plots, representing three independent experiments (data in S1 Table).

To assess the sensitivity of PCR using the DY682 or digoxigenin labeled primer compared to ethidium bromide labeling, the amount of genomic DNA was varied, as shown in Fig. 2. The band intensities obtained by the DY682 and digoxigenin labeled primer showed strong linear relations with the log of the DNA dose (r^2^ values of 0.99), whereas ethidium bromide-labeled band intensities showed less linearity. Furthermore, the standard PCR/ethidium bromide method was able to detect a specific PCR product with as little as 1.3 ng gDNA, whereas the DY682 and digoxigenin-labeled primers enabled detection of the PCR product with only 0.02 ng of DNA, providing an approximately 60-fold increase in sensitivity.

### Comparative accuracy of α:β tryptase genotype determination by PCR/EcoRV

**Figure 3:**
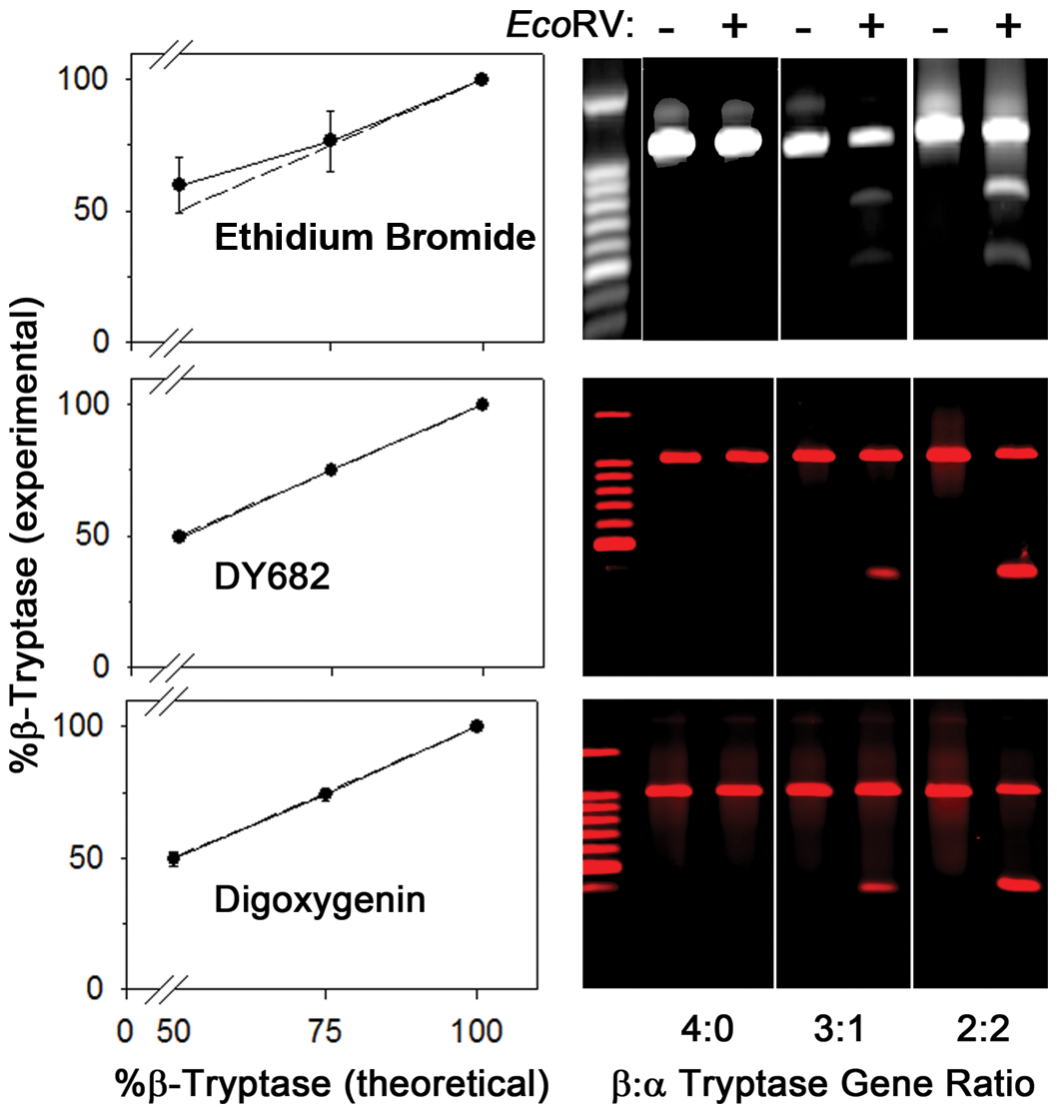
α/β-Tryptase genotyping using EcoRV-digested amplimers. EcoRV-digested amplimers were labeled with ethidium bromide after gel electrophoresis (top panels) or, during the final PCR cycle with DY682 (middle panels) or digoxigenin (bottom panels) labeled 3′-primer. gDNA from SMCs with known ββββ, βββα and ββαα genotypes, defined as 4∶0, 3∶1 and 2∶2 β:α ratios, were utilized. Experimentally calculated percentages of the β-tryptase gene were plotted against the known percentages of β-tryptase (n = 3, mean ± SD). Standard deviations were too small to visualize the error bars in the 50 and 75%β-Tryptase (theoretical) values for the middle plot. Data is stored in S2 Table.

The accuracy of the three genotyping methods was compared in Fig. 3 using gDNA from SMCs with β:α tryptase gene ratios of 4∶0 (ββββ), 3∶1 (βββα), and 2∶2 (ββαα). Using 3′-labeled primers, experimental percentages of β-tryptase genes were essentially identical to those predicted, whereas after ethidium bromide labeling the experimental percentages deviated upward from the predicted percentage of the β-tryptase genes when the genotype was 2∶2 (ββαα), and exhibited greater variability at both the 2∶2 and 3∶1 ratios such that the mean percentages at these ratios were not significantly different than one another (p = 0.14, Student t-test). The actual measured percentages of β-tryptase genes for known β:α tryptase gene ratios of 2∶2; 3∶1 and 4∶0, respectively, using DY682-labeled primer were 49.6±0.7, 75.1±0.6, and 100±0, using digoxigenin-labeled primer were 49.6±2.5, 74.1±2.4 and 100±0, and using ethidium bromide labeling were 59.0±10.8, 76.1±11.9 and 100±0. In contrast to the comparison of gene ratios at 3∶1 and 2∶2 with ethidium bromide-labeled bands, which were not significantly different from one another (p = 0.06), those with DY682- or digoxigenin- labelled bands were each significantly different than one another (p<0.001) by ANOVA. Each of the three techniques enabled distinction between 3∶1 and 4∶0 genotypes (p<0.04 for ethidium bromide; p<0.001 for DY682 and digoxigenin). Because the *α-* and β-tryptase genes are 93% homologous, heteroduplexes of *α/β-*tryptase gene products likely formed during the annealing phase of PCR, and these heteroduplexes are resistant to EcoRV enzymatic digestion, leading to a higher apparent level of β-tryptase genes.

### Using enhancers to improve the sensitivity of PCR of α/β-tryptase genes

**Figure 4:**
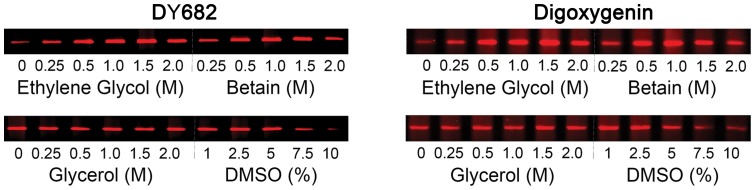
A comparison of potential enhancers on the PCR of α/β-tryptase gDNA using DY682-labeled (left panels) or digoxigenin-labeled (right panels) 3′-primer. The effects of ethylene glycol (0.5 to 2.5 M), betaine (0.5 to 2.5 M), glycerol (0.5 to 2.5 M), DMSO (0.5 to 10%) or no additive was examined on PCRs performed with gDNA amounts of 0.3-0.5 ng and Taq DNA polymerase of 0.75 U per PCR with ethylene glycol or betaine, or 1.2–2.0 ng of gDNA and 1.25 U of Taq DNA polymerase per PCR with glycerol or DMSO. Representative images of the 1017-28 bp amplimers from three independent experiments are shown. Band intensity data is stored in S3 Table.

The %GC content of the amplified tryptase gene sequences is about 68%, which can decrease the PCR efficiency. To optimize PCR in the current study, several additives were examined, including ethylene glycol, betaine, glycerol and DMSO, as described for PCR of other GC-rich DNA domains [26]–[29]. Ethylene glycol or betaine facilitate strand separation by altering the DNA melting characteristics and increasing the resistance of *Taq* polymerase to denaturation, while DMSO weakens hydrogen bonding, improving the specificity of primer annealing and preventing premature terminations due to intra and inter strand annealing [28], [29]. To determine at what concentration these additives optimized the generation of full-length amplimers from α/β-tryptase genes using gDNA from SMCs or MAC-6 cells, DMSO (1 to 10%) and ethylene glycol (0.5–2.0 M), betaine (0.5–2.0 M) and glycerol (0.5–2.0 M) conditions were examined, as shown in Fig. 4. Ethylene glycol (0.5–1.5 M) and betaine (0.5–1.5 M) greatly and comparably improved the efficiency of amplification of the 1017-28 bp tryptase gene products, 1 M ethylene glycol by 2.9±0.5 fold for DY682 and 2.5±0.2 fold for digoxigenin and 1 M betaine by 2.9±0.3 fold for DY682 and 2.6±0.3 for digoxygenin, calculated as the band signal intensity ratio (‘with’ over ‘without’ each enhancing agent, mean ± STD) using the Odyssey system. DMSO inhibited the PCR amplification at high concentrations, whereas neither DMSO nor glycerol had an apparent enhancing effect. Thus, ethylene glycol and betaine were the most effective PCR enhancers, and 1.0 M betaine was chosen for the standard method.

### Genotyping of individuals

**Figure 5:**
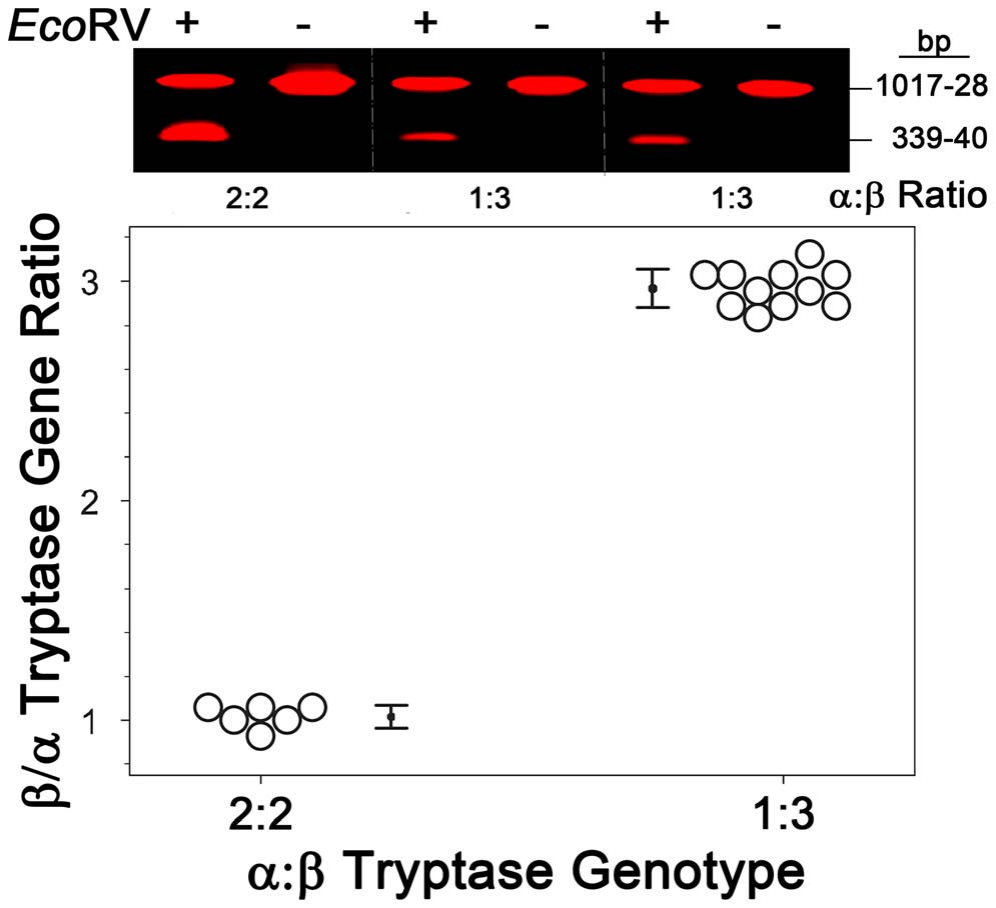
Tryptase genotyping in healthy subjects. α/β-Tryptase genotyping was performed with gDNA obtained from SMCs of 24 subjects. EcoRV digestions of PCR amplimers labeled during the final cycle with DY682-labeled 3′-primer were performed. Gel images are shown for 3 of the 24 samples analyzed, 2 with a β:α ratio of 3∶1 and 1 with a ratio of 2∶2. The 17 samples in which the α-tryptase gene was detected are shown in the plot, where those with a 2∶2 or 3∶1 genotype are grouped together. Data is stored in S4 Table.

PCR using DY682-labelled primer was performed to investigate the α/β tryptase genotype on 24 subjects, as shown in Fig. 5. Subjects grouped into the 2∶2 and 1∶3 α:β-tryptase genotypes showed no overlap, mean ± STD (range) values being 1.02±0.05 (0.93–1.07) and 2.97±0.09 (2.8–3.1), respectively, and means were significantly different (P<0.001).All subjects had β-tryptase gene amplimers, while 17 of the subjects also had an α-tryptase gene. The frequency of the α-tryptase allele (α_f_) at the single locus where this gene resides was 0.52, whereas the overall frequency of the α-tryptase allele at both α/β-tryptase loci (α′_f_) was 0.26; yielding an overall β′_f_ value of 0.74. The respective 2∶2, 3∶1 and 4∶0 β:α tryptase gene ratios were 0.25, 0.46, and 0.29, showing Hardy-Weinberg Equilibrium (Χ^2^ = 0.62; P = 0.43 with 1 degree of freedom), [30] consistent with prior reports [16], [17].

## Discussion

PCR of homologous α/β-tryptase genes, using a primer set that recognizes both alleles, results in a mixture of homo- and hetero- duplexes. Using EcoRV to discriminate the α-tryptase amplimer, containing a susceptible restriction site, from the β-tryptase amplimer, having no such restriction site, can be problematic due to the formation of α/β-tryptase heteroduplexes as α and β- tryptase amplimers can anneal to one another, and these heteroduplexes lack susceptibility to cleavage by this restriction enzyme. By utilizing a labeled primer during the final PCR cycle, the labeled amplimers contain only homoduplexes of α or β tryptase gene products. This most likely explains why ethidium bromide labeling failed to discriminate between αβ:αβ (2∶2) and αβ:ββ (1∶3) tryptase genotypes (Fig. 3); variable formation of α/β-tryptase heteroduplexes likely increased the apparent amount of the β gene product. In contrast, the 2∶2 and 3∶1 β:α genotypes were discriminated from one another by both the DY682 and the digoxigenin techniques (Fig. 3 and 5). Also, the DY682- or digoxigenin- labeled 3′ primer together with infrared fluorescent signaling as employed in the current study resulted in a very low background and a strong fluorescent signal, improving sensitivity compared to ethidium bromide labeling of amplimers and their fragments by about 60-fold, while avoiding the hazards of working with radioactivity. However, because the fluorescence of DY682-labeled products could be assessed directly in the gel, while detection of digoxigenin-labeled products required blotting followed by immunolabeling, the former takes only a few hours to perform and was considered the best technique. Thus, the PCR/restriction enzyme technique for determining the α/β-tryptase genotype is sensitive, accurate, efficient and safe, and should permit high throughput screening to detect potential phenotypes due to the presence of the α-tryptase gene, and be applicable to analogous situations with other closely related alleles.

## Supporting Information

S1 Table **Fig. 2** **data.**
(PDF)

S2 Table **Fig. 3** **data.**
(PDF)

S3 Table **Fig. 4** **data.**
(PDF)

S4 Table **Fig. 5** **data.**
(PDF)
