# Supplementary material for: A Simple, Sensitive and Safe Method to Determine the Human α/β-Tryptase Genotype
Source: PLoS One. 2014 Dec 29;9(12):e114944. doi: 10.1371/journal.pone.0114944 (PMC4278853; doi:10.1371/journal.pone.0114944)
Supplement: S1 Table — Fig. 2 data. (PDF) [file pone.0114944.s001.pdf]

Table S1.
Figure 2 data.

DNA(ng)	SMC (DY682)	Mac6 (DY6	SMC (digoxygenir	Mac6 (digo	SMC (Ethidium Br)	Mac6 (Ethidium Br)
0.02	13	11.5	7.7	5		
0.08	27	26.8	17	12		
0.3199	43	48.5	34	27	2	1.29
1.2797	54	63	48	42	28	7.5
5.1188	65	75	60	52	54	12.5
20.475	72	92	73	70	75	35
81.9	87	107	83	83	151	65.5
