# Supplementary material for: A Simple, Sensitive and Safe Method to Determine the Human α/β-Tryptase Genotype
Source: PLoS One. 2014 Dec 29;9(12):e114944. doi: 10.1371/journal.pone.0114944 (PMC4278853; doi:10.1371/journal.pone.0114944)
Supplement: S2 Table — Fig. 3 data. (PDF) [file pone.0114944.s002.pdf]

**Table S2.**
**Figure 3 data.**

| DY682 (IRD) PCR | | 3:1 Band intesities | %β/(α+β) | 2:2 Band intesities | %β/(α+β) |
|---|---|---|---|---|---|
| Exp 1 | β | 30.2 | 75.88% | 28.3 | 48.56% |
| | α | 9.6 | | 29.98 | |
| Exp 2 | β | 27.7 | 74.66% | 23.6 | 49.79% |
| | α | 9.4 | | 23.8 | |
| Exp 3 | β | 56 | 74.67% | 40.3 | 49.81% |
| | α | 19 | | 40.6 | |
| Exp 4 | β | 64 | 75.29% | 39.65 | 50.09% |
| | α | 21 | | 39.5 | |

| | mean% | STD |
|---|---|---|
| 3:1 | 100 | 0 |
| 2:2 | 75.13 | 0.58 |
| | 49.56 | 0.68 |

(P = <0.001; 4:0 v 3:1, 3:1 v 2:2)
All Pairwise ANOVA, Holm-Sidak post hoc test

| Digoxygenin PCR | | 3:1 Band intesities | %β/(α+β) | 2:2 Band intesities | %β/(α+β) |
|---|---|---|---|---|---|
| Exp 1 | β | 28.3 | 71.83% | 25.3 | 52.54% |
| | α | 11.1 | | 22.85 | |
| Exp 2 | β | 68.5 | 76.54% | 56.6 | 48.13% |
| | α | 21 | | 61 | |
| Exp 3 | β | 50.7 | 74.01% | 39 | 48.21% |
| | α | 17.8 | | 41.9 | |

| | mean% | STD |
|---|---|---|
| 3:1 | 100 | 0 |
| 2:2 | 74.13 | 2.36 |
| | 49.63 | 2.53 |

(P = <0.001; 4:0 v 3:1, 3:1 v 2:2)
All Pairwise ANOVA, Holm-Sidak post hoc test

| | | | | | | | | | | | |
|---|---|---|---|---|---|---|---|---|---|---|---|
| Ethidium Bromide PCR | | | | | | | | | | mean% | STD |
| Exp 1 | | β | 952 | | 73.73% | 1344 | | 65.67% | 3:1 | 100 | 0 |
| | | α | 112 | 339 | | 232 | 702 | | 2:2 | 76.05 | 11.87 |
| | | | | | | | | | | 58.95 | 10.75 |
| Exp 2 | | β | 690 | | 65.50% | 1477 | | 46.55% | | (P = 0.064; 3:1 vs 2:2) | |
| | | α | 120 | 363 | | 560 | 1696 | | | (P=0.038; 4:0 vs 3:1) | |
| | | | | | | | | | All Pairwise ANOVA, Holm-Sidak post hoc test | | |
| Exp 3 | | β | 1384 | | 88.91% | 1626 | | 64.62% | | | |
| | | α | 57 | 173 | | 294 | 890 | | | | |
| | | BP | | | | | | | | | |
| | β | | 1028 | | | | | | | | |
| | α | | 339.5 | | | | | | | | |
