# Supplementary material for: A Simple, Sensitive and Safe Method to Determine the Human α/β-Tryptase Genotype
Source: PLoS One. 2014 Dec 29;9(12):e114944. doi: 10.1371/journal.pone.0114944 (PMC4278853; doi:10.1371/journal.pone.0114944)
Supplement: S3 Table — Fig. 4 data. (PDF) [file pone.0114944.s003.pdf]

Table S3.
Figure 4 data.

DY682 PCR

Exp#	**Ethylene glycol**							
#1	**none**		**0.25**	**0.5**	**1**	**1.5**	**2M**	
		13.3	19.66	32	33.9	38	28	band intensity
					2.548872	ratio		
#2	**none**		**0.25**	**0.5**	**1**	**1.5**	**2**	
		7.7	11.5	19.8	21	24	19.8	band intensity
					2.727273	ratio		
#3	**none**		**0.25**	**0.5**	**1**	**1.5**	**2M**	
		5.6	13.5	17	19.5	24.4	11.2	band intensity
					3.482143	ratio		
	**Betaine**							
#1	**none**		**0.25**	**0.5**	**1**	**1.5**	**2 M**	
		13.3	19.5	30	35	27.9	18.9	band intensity
					2.631579	ratio		
#2	**none**		**0.25**	**0.5**	**1**	**1.5**	**2**	
		7.7	13	18	22.3	18.4	10.9	band intensity
					2.896104	ratio		
#3	**none**		**0.25**	**0.5**	**1**	**1.5**	**2**	
		5.6	8.6	13.6	18.5	22.2	16	band intensity
					3.303571	ratio		

	**Glycerol**						
#1	**None**	**0.25**	**0.5**	**1**	**1.5**	**2M**	
	23	22.8	21,3	22.9	20.3	23.9	band intensity
				0.995652	ratio		
#2	**None**	**0.25**	**0.5**	**1**	**1.5**	**2M**	
	11.8	11	11	12.1	11	13.8	band intensity
				1.025424	ratio		
#3	**None**	**0.25**	**0.5**	**1**	**1.5**	**2M**	
	36.3	33	32	37	32	36	band intensity
				1.019284	ratio		
	**DMSO**						
#1	**none**	**1**	**2.5**	**5**	**7.5**	**10%**	
	23	24.9	24.55	28.26	12.4	6	band intensity
				1.228696	ratio		
#2	**none**	**1**	**2.5**	**5**	**7.5**	**10%**	
	11.8	13.2	15	15.7	7.3	2.9	band intensity
				1.330508	ratio		
#3	**none**	**1**	**2.5**	**5**	**7.5**	**10%**	
	36.3	41	47	47	29	18	band intensity
				1.294766	ratio		

Digoxygenin PCR

Ethylene glycol

#1	**none**	**0.25**	**0.5**	**1**	**1.5**	**2M**	
	17	26	43.4	45	55	38.8	band intensity
				2.647059	ratio		
#2	**none**	**0.25**	**0.5**	**1**	**1.5**	**2M**	
	22.3	36	53	57.3	64.6	42.4	band intensity
				2.569507	ratio		
#3	**none**	**0.25**	**0.5**	**1**	**1.5**	**2M**	
	23.5	40.4	50	54	60	46.7	band intensity
				2.297872	ratio		

Betaine

#1	**none**	**0.25**	**0.5**	**1**	**1.5**	**2 M**	
	17	25	41	50	37	28	band intensity
				2.941176	ratio		
#2	**none**	**0.25**	**0.5**	**1**	**1.5**	**2 M**	
	22.3	25.5	32.2	55	40	34	band intensity
				2.466368	ratio		
#3	**none**	**0.25**	**0.5**	**1**	**1.5**	**2 M**	
	23.5	32	37	56	30	22.6	band intensity
				2.382979	ratio		

Glycerol

#1	**None**		**0.25**	**0.5**	**1**	**1.5**	**2M**
		30	27.6	28.2	29.4	28.3	32 band intensity
					0.98 ratio		
#2	**None**		**0.25**	**0.5**	**1**	**1.5**	**2M**
		15.7	14.5	16.2	18	18	17.6 band intensity
					1.146497 ratio		
#3	**None**		**0.25**	**0.5**	**1**	**1.5**	**2M**
		11	11	10	11	10	11 band intensity
					1 ratio		

DMSO

#1	**none**		**1**	**2.5**	**5**	**7.5**	**10%**
		30	37.3	34.5	27.6	16	14 band intensity
					0.92 ratio		
#2	**none**		**1**	**2.5**	**5**	**7.5**	**10%**
		15.7	18	19	18.2	14.4	6 band intensity
					1.159236 ratio		
#3	**none**		**1**	**2.5**	**5**	**7.5**	**10%**
		11	11	11.4	11.8	8.1	55 band intensity
					1.072727 ratio		

	1 M Ethylene Glycol		1 M Betaine		1 M Glycerol		5% DMSO	
	avg	std	avg	std	avg	std	avg	std
DY682	2.92	0.50	2.94	0.34	1.01	0.02	1.28	0.05
Digoxygenin	2.50	0.18	2.60	0.30	1.04	0.09	1.05	0.12
