# Supplementary material for: A Simple, Sensitive and Safe Method to Determine the Human α/β-Tryptase Genotype
Source: PLoS One. 2014 Dec 29;9(12):e114944. doi: 10.1371/journal.pone.0114944 (PMC4278853; doi:10.1371/journal.pone.0114944)
Supplement: S4 Table — Fig. 5 data. (PDF) [file pone.0114944.s004.pdf]

Table S4.
Figure 5 data.

Subject #		1	2	3	4	5	6	7	8	9
Band	1028 bp	37.1	33.8	31.9	34	40.8	36.8	38.6	30.6	29.7
	339.5 bp	12.5	11.7	32.2		13.5	12.5			27.8
1028 bp/339.5 bp		**2.97**	**2.89**	**0.99**	**4:0**	**3.02**	**2.94**	**4:0**	**4:0**	**1.07**

Subject #		10	11	12	13	14	15	16	17	18
Band	1028 bp	32.7	49.1	40.7	41.8	53	43.3	45.4	25.3	50
	339.5 bp	35.3		14.1	13.7		41.14	43.2	8.1	
1028 bp/339.5 bp		**0.93**	**4:0**	**2.89**	**3.05**	**4:0**	**1.05**	**1.05**	**3.12**	**4:0**

Subject #		19	20	21	22	23	24
Band	1028 bp	82.5	87.7	61.6	101	86.5	86
	339.5 bp	29.1	29	61.2		30	28.5
1028 bp/339.5 bp		**2.84**	**3.02**	**1.01**	**4:0**	**2.88**	**3.02**

ββββ	ßßαα	ßßßα
4	0.991	2.968
4	1.068	2.889
4	0.926	3.022
4	1.053	2.944
4	1.051	2.887
4	1.007	3.051
4		3.123
		2.835
		3.024
		2.883
		3.018

	ββββ	ßßαα	ßßßα
mean	4	1.016	2.968
std	0	0.05303	0.08828
min		0.926	2.835
max		1.068	3.123
T test		2.76E-18	
(2:2 v 1:3)			
